# Expression of taste receptors in Solitary Chemosensory Cells of rodent airways

**DOI:** 10.1186/1471-2466-11-3

**Published:** 2011-01-13

**Authors:** Marco Tizzano, Mirko Cristofoletti, Andrea Sbarbati, Thomas E Finger

**Affiliations:** 1 Rocky Mountain Taste and Smell Center, Department of Cell and Developmental Biology, University of Colorado at Denver & Health Sciences Center, Aurora, USA; 2 Department of Morphological-Biomedical Sciences, University of Verona, Verona, Italy

## Abstract

**Background:**

Chemical irritation of airway mucosa elicits a variety of reflex responses such as coughing, apnea, and laryngeal closure. Inhaled irritants can activate either chemosensitive free nerve endings, laryngeal taste buds or solitary chemosensory cells (SCCs). The SCC population lies in the nasal respiratory epithelium, vomeronasal organ, and larynx, as well as deeper in the airway. The objective of this study is to map the distribution of SCCs within the airways and to determine the elements of the chemosensory transduction cascade expressed in these SCCs.

**Methods:**

We utilized a combination of immunohistochemistry and molecular techniques (rtPCR and in situ hybridization) on rats and transgenic mice where the Tas1R3 or TRPM5 promoter drives expression of green fluorescent protein (GFP).

**Results:**

Epithelial SCCs specialized for chemoreception are distributed throughout much of the respiratory tree of rodents. These cells express elements of the taste transduction cascade, including Tas1R and Tas2R receptor molecules, α-gustducin, PLCβ2 and TrpM5. The Tas2R bitter taste receptors are present throughout the entire respiratory tract. In contrast, the Tas1R sweet/umami taste receptors are expressed by numerous SCCs in the nasal cavity, but decrease in prevalence in the trachea, and are absent in the lower airways.

**Conclusions:**

Elements of the taste transduction cascade including taste receptors are expressed by SCCs distributed throughout the airways. In the nasal cavity, SCCs, expressing Tas1R and Tas2R taste receptors, mediate detection of irritants and foreign substances which trigger trigeminally-mediated protective airway reflexes. Lower in the respiratory tract, similar chemosensory cells are not related to the trigeminal nerve but may still trigger local epithelial responses to irritants. In total, SCCs should be considered chemoreceptor cells that help in preventing damage to the respiratory tract caused by inhaled irritants and pathogens.

## Background

Chemical irritation of the respiratory and tracheal mucosa causes various reflex responses such as coughing and apnea. Similarly, chemical stimulation of the larynx results in a number of protective reflexes involved in respiratory regulation, including startle, swallowing, apnea, laryngeal constriction, hypertension, and bradycardia [1-7]. Such disturbance of respiration, if prolonged, may cause profound hypoxemia and even death [8,9]. Despite obvious physiological and clinical importance, not enough information is available regarding the means by which chemical irritants are detected.

Until recently, the presumption has been that while taste buds may mediate chemical detection in the epiglottis [2], free nerve endings are responsible for detection of irritant chemicals lower in the respiratory tract [7]. Within the last decade, researchers have identified a population of specialized chemoreceptive epithelial cells scattered along most of the respiratory tract from nasal cavity to bronchi [10-18]. These so-called solitary chemosensory cells (SCCs) were first described in the gill apparatus and skin of aquatic vertebrates [19] and were identified by having a slender apical process and substantial basolateral contacts with nerve fibers suggesting their role as sensory elements.

The chemosensitive free nerve endings and SCCs of the airways utilize different receptors and therefore are responsive to different chemical irritants [20]. The nerve endings utilize various TRP channels (e.g. TrpV1 [21,22] or TrpA1 [23]), ASICs [24] and other chemosensitive ion channels. In contrast, the SCCs rely on taste receptors and their related downstream signalling cascade to activate the system: the G-protein, α-gustducin; the phospholipase C beta2 (PLCβ2); and the transient receptor potential channel M5 (TrpM5) [10-17].

The population of SCCs within the airways has been identified by expression of the TrpM5 channel [12,13,20,25]. Functional studies in TrpM5-knockout mice show that activity of the TrpM5 channel is necessary for chemical transduction in both taste and SCCs [20,26,27]. The SCCs of the upper and lower airways are structurally diverse and have different relationships to nerve fibers. In the nasal cavity, the SCCs have loose apical microvilli but are intimately associated with and synapse onto sensory nerve terminals of the trigeminal nerve. In contrast, some of the SCCs of the lower airways have the key characteristics of brush cells [28] including the apical tuft of stiff microvilli. Despite these differences in morphology, both the nasal SCCs and the tracheal SCC brush cells utilize the chemoreceptive transduction cascade first described in taste buds. Likewise, the SCCs in fish epidermis utilize some of the same receptor proteins as in the taste system [29].

The gustatory system uses different families of taste receptors to detect nutritive or beneficial (sweet/umami) compounds on the one hand and potentially harmful (bitter) substances on the other. The appetitive qualities (sweet and umami) are detected via GPCRs of the Tas1R family, namely Tas1R1, Tas1R2, and Tas1R3, characterized by a long extracellular NH2-terminal segment [30-32]. Bitter substances are detected via GPCRs of the numerous members of the Tas2Rs family [33], characterized by a short extracellular NH2-domain [34-37]. The members of the Tas1R family of taste receptors function as heterodimers [34]: the Tas1R2/Tas1R3 complex binds sweet-tasting stimuli, whereas Tas1R1/Tas1R3 binds amino acids. Thus Tas1R3 is an obligatory subunit for both of the appetitive quality taste receptors [31,38,39].

Recently, it has been reported that most of the SCCs in the nasal epithelium express Tas2Rs [10,40] with a smaller number expressing Tas1R3 (using a Tas1r3-WGA transgenic mice). Although the SCCs express taste receptor proteins, the sensations elicited by chemical stimulation of the SCCs are not tastes, but rather one of pain or irritation. This follows from the fact that SCCs synapse onto polymodal nociceptors of the trigeminal nerve rather than taste nerves [10,41] and it is the nerves rather than the receptors that dictate the quality of a sensation [42]. Our recent studies indicate that SCCs in the nasal cavity are activated by a variety of substances [13,25], including bacteria quorum sensing signalling molecules [20] and trigger protective airway reflexes e.g. respiratory depression and apnea [10,20]. Chemical stimulation of the larynx results in additional protective reflexes including startle, swallowing, laryngeal constriction, hypertension, and bradycardia [1]. Lower in the airways, chemical irritation largely triggers cough.

To better understand the airway SCC system and its receptors in two common laboratory rodents, we investigated the distribution of SCCs throughout the lower airways - from larynx to lung. The results show that SCCs decrease in prevalence as one descends in the respiratory tract and that the two different classes of taste receptors are differentially expressed in upper and lower airways.

## Methods

### Animals

Adult transgenic mice in which either the Tas1R3 or TrpM5 promoter drives expression of GFP were used. Animals were a gift of Robert F. Margolskee (currently of Monell Chem Senses Ctr., Philadelphia, PA) and Sami Damak (currently of Nestle, Lausanne, Switzerland). The Tas1R3-GFP construct contained 5' to 3': 13 kb of the mouse Tas1R3 gene including the 5' flanking region and the entire 5' untranslated region, and the coding sequence for eGFP [43]. The TrpM5-GFP construct contained 5' to 3': 11 kb of mouse TrpM5 5' flanking sequence, TrpM5 Exon 1 (untranslated), Intron 1, and the untranslated part of Exon 2, and eGFP [43]. Adult Wistar rats were used for the in situ hybridization (ISH) and reverse trascriptase-polymerase chain reaction (RT-PCR) experiments.

Experiments on mice were undertaken with the approval of Univ. Colorado Denver Inst. Animal Care and Use Comm. Experiments on rats were conducted in accordance with the guidelines for animal experimentation according to Italian law.

### Whole mount fluorescence

The nose and trachea were dissected from Tas1R3- and TrpM5-GFP transgenic mice and stored in Tyrode's buffer (145 mM NaCl, 5 mM KCl, 1 mM CaCl_2_, 1 mM MgCl_2_, 1 mM Na-pyruvate, 20 mM HEPES, 5 mM glucose, 7.2 pH with NaOH) to preserve the eGFP fluorescence. Micrographs of whole-mounted tissues were captured with a RT Slider Spot Camera (Diagnostic Instruments) connected to a stereo microscope Olympus SZX12 (Olympus Corporation).

### Immunofluorescence

For histological studies, tissue from Tas1R3-GFP and TrpM5-GFP transgenic mice was dissected after perfusion-fixation in 4% PFA/0.1M phosphate buffer (PB: 25 mM sodium phosphate dibasic anhydrous, 75 mM sodium phosphate monobasic monohydrate; pH 7.2) and postfixed in the same fixative for 30 minutes followed by cryoprotection in 20% sucrose in 0.1 M PB pH7.2 overnight at 4°C. After sectioning transversely on a cryostat, 16-μm sections were collected and dried onto Superfrost Plus slides (Fisher Scientific; USA). After three times 10 min washes in 0.1 M phosphate-buffered saline (PBS: 150 mM sodium cloride, 25 mM sodium phosphate dibasic anhydrous, 75 mM sodium phosphate monobasic monohydrate; pH 7.2), slides were incubated in blocking solution (2% normal goat serum, 1% bovine serum albumin, 0.3% Triton in PBS) for 1 hour at RT. Incubation with rabbit (rb) anti-α-gustducin antibody (1:500) (catalog # sc-395, Santa Cruz Biotechnology, USA), anti-Plcβ2 (1:1000) (catalog # sc-206, Santa Cruz Biotechnology, USA), anti PGP9.5 (1:500) (catalog # 7863-0504, AbD Serotec, USA), anti-CGRP (1:1000) (catalog # T-4032, Peninsula Laboratories LLC, USA), anti-TrpM5 antibody (1:2,000) (Emily R. Liman, Univ. Southern California, USA) and rat anti-SubP (1:1000) (catalog # YMC1021, Accurate Chemical & Scientific Company, NY, USA) all diluted in blocking solution was carried out overnight. Three PBS washes were followed by 2 hours of incubation with Alexa568 goat anti-rb or goat anti-rat (1:400; Molecular Probes, USA). The slides then were washed one time for 10 minutes in 0.1 M PB and two times for 10 minutes in 0.1 M PBS before coverslipping slides with Fluormount G (Southern Biotechnology Associates, USA). Omission of the primary antibody resulted in no apparent fluorescent signal. Similarly, in wild type mice no significant autofluorescence is apparent (Additional file 1) at GFP wavelengths, thus indicating specificity of the fluorescence in the GFP transgenic lines.

All images were collected with an Olympus Fluoview confocal laser scanning microscope (LSCM) FV300 (Olympus Corporation). For each image, the channels were collected sequentially with single wavelength excitation and then merged to produce the composite image using the Fluoview v5.0 software. This avoids the problem resulting from side-band excitation of the fluorochromes. Brightness and contrast were adjusted in Adobe Photoshop.

### Total RNA isolation and Reverse trascriptase - polymerase chain reaction (RT-PCR)

Experimental tissues were dissected rapidly from 10 Wistar rats and frozen on dry ice. RNA was isolated from taste tissue (vallate papillae, foliate papillae, fungiform papillae) and from other rat tissues (heart, trachea, bronchi, and lungs, nasal respiratory epithlium, larynx, spleen, liver, gut, stomach, testicle, brain) using TRIzol Reagent (Invitrogen, life technologies). Samples of RNA (about 1 μg of total RNA) were digested with DNase I, Amp Grade (Invitrogen, life technologies), reverse transcribed and amplified with gene-specific primers using the SuperScript First-Strand Synthesis System for RT-PCR kit (Invitrogen, life technologies). Controls omitting reverse transcriptase were done and were negative (Additional file 2). Primer sequences used to amplify the target genes are shown in Table1. Expression of GAPDH (Glyceraldehyde-3 phosphate dehydrogenase) was used as the internal standard (Additional file 2). Amplification was performed with HotMaster Taq (Eppendorf) in an Eppendorf Gradient Martercycler at 95°C × 30 sec, at 57-61°C × 30 sec, and at 72°C × 45-90 sec for 30 cycles. PCR products were visualized with ethidium bromide on a 1.5% agarose gel by electrophoresis. Sequencing and nested PCR with 2 internal primers of the PCR products were used to confirm to specificity of the PCR and the primers used (data not shown). To check the quality of the cDNA templates used for all the PCR experiments we amplified with primers for GAPDH the cDNA treated with and without (Additional file 2) the reverse transcriptase (RT) enzyme.

**Table 1:** List of the primer sequences as used for the RT-PCR and ISH experiments.

Gene	UniGene ID	Primer	Sequence primer 5'-3'	Annealing T °C
**α-gustducin**	Rn.10456	Forward	CTG CTC TGA CGA TCT ATC TC	57

		Reverse	GGT CAC TTA CAG CTC ACT TC	

**Tas1R1**	Rn.92309	Forward	CGG TTC ACT GTT GAG GAG AT	57.4

		Reverse	CCT GAA GAA CAC TCT AGC CA	

**Tas1R2**	Rn.222086	Forward	CAG TTC TGC ATA ACC TCA CG	55.5

		Reverse	CTT GTA GGA CCA CAT GGA AC	

**Tas1R3**	Rn.81025	Forward	AGT TGC TAC GCC AAG TGA AC	56.5

		Reverse	AGG TGA AGT CAT CTG GAT GC	

**Tas2R119**	Rn.48782	Forward	GTC ATT GTC GTT GTC CAT GC	60

		Reverse	CTT CTG AGC AGG ATG TCT TG	

**Tas2R121**	Rn.48786	Forward	TTA GTC TCT GGC TTG CCA CC	60.9

		Reverse	AGA GTA AGA GGA AGG AGA CC	

**Tas2R107**	Rn.48784	Forward	CAT TCT CAT TGG CTT GGC GA	55.2

		Reverse	TTA AGT GCT GCA GTG CCT TC	

**Tas2R13**	Rn.48787	Forward	TAG TCA CTT CAG CCT CTG GT	58.3

		Reverse	TAG AGC AAG AGG AAG GAG AC	

**Tas2R123**	Rn.48792	Forward	CAT GGA CTG GCT CAA GAG GA	60.2

		Reverse	CTA AGA CAA GGC AGC ACA GA	

**Tas2R105**	Rn.48788	Forward	GCC AAG AAC AAG AAG CTC TC	53.9

		Reverse	GGA TAG ACG GAT GCA GTT GT	

**Tas2R134**	Rn.143008	Forward	GTG ACA TGA TTG TGG CTT GC	55.2

		Reverse	CGC CTC TTG TCT TGT GAT CT	

**Tas2R126**	Rn.48794	Forward	CCT CAG ACA TGA TCC TCC TC	59

		Reverse	GTG CCT CGG AAC TTG AGA TT	

**GAPDH**	Rn.91450	Forward	ACT GGC GTC TTC ACC ACC AT	61

		Reverse	ATC CAC AGT CTT CTG GGT GG	

### In situ Hybridization (ISH)

A longer cDNA insert (1.43 kb) for rat α-gustducin and a cDNA insert (1.85 kb) for rat Tas1R3 were generated by RT-PCR, using cDNA from rat lingual tissue mRNA as template (primers in Table 1). This sequence included the entire coding sequence (McLaughlin et al. 1992) of α-gustducin and part of the extracellular domain and the entire transmembrane sequences of Tas1R3. RT-PCR products were cloned using the TOPO TA Cloning kit (Invitrogen life technologies, USA) into the pCRII-TOPO vector. Digoxigenin-labeled RNA (DIG-RNA) probes were transcribed in anti-sense and sense orientation using T7 and SP6 RNA polymerase. To estimate probe concentration, serial dilutions of labeled probes and a standard labeled RNA were spotted on nylon membranes (HybondN^+^; Amersham, USA), immunodetected using anti-DIG-Fab-AP conjugate diluted 1:5000, and visualized with NBT and BCIP according to instructions from Roche. Probes were diluted to10 ng/μl and were stored in aliquots at -80°C.

Tissues were dissected rapidly from adult Wistar rats after perfusion with 0.1 M PB and fixation with 4% PFA/0.1M PB. The tissues were postfixed and cryoprotected overnight at 4°C in 4% PFA/0.1M PB + 20% sucrose. For preparing cryosections, tissues were placed in chilled OCT embedding medium and snap-frozen in isopentane precooled in dry ice. Tissue blocks were stored at -80C for up to 4 weeks. Cryosections of 10-12 μm were cut at -20°C, collected on baked Superfrost Plus slides (Fisher Scientific; USA), and stored desiccated at -80°C. Cryosections were removed from -80°C storage and were immediately fixed in freshly prepared 4% PFA/0.1M PBS at 4°C for 20 minutes. Sections were rinsed twice in PBS for 5 min each. Endogenous AP activity was quenched with 0.2 M HCl for 8 minutes, followed by two 5-min washes in PBS. For tissue partial proteolysis, sections were permeabilized with 10 μg/ml proteinase K in 10 mM Tris-HCl (pH 7.5) at room temperature (RT) for 10 minutes (lingual tissue). After rinsing in 10 mM Tris-HCl, sections were equilibrated in 0.1 M triethanolamine (TEA) for 2 minutes and then were acetylated in freshly prepared 0.25% acetic anhydride in 0.1 M TEA for 10 minutes. After rinsing in 2× SSC for 10 minutes at RT, sections were air-dried for 5 minutes on a slide warmer at 60°C. Sections were encircled with rubber cement to enclose hybridization buffer and were used immediately in hybridization. DIG-RNA probes were freshly diluted in 1 ml hybridization buffer (50% formamide, 2× SSC, 1× Denhardt's, 10% dextran sulphate, 0,5 mg/ml yeast tRNA, 0.5 mg/ml salmon sperm DNA), denatured at 95°C for 5-10 minutes and cooled on ice for 2 minutes, all steps in the dark (since digoxigenin is light-sensitive). Probe in hybridization buffer (400 μl/slide) was directly applied to dry preheated sections. Slides were incubated overnight in a humid chamber containing paper towels moistened with 50% formamide and 2× SSC in the dark. The temperature for hybridization and post-hybridization high stringency washes were 59°C for α-gustducin and 57-60°C for Tas1R3. Sections were washed in 2× SSC at hybridization temperature for 10 minutes to remove excess probe. Sections were then subjected to two high stringency washes in 50% formamide/1× SSC at the hybridization temperature for 20 minutes each time. Slides were rinsed in wash buffer (100 mM maleic acid, 150 mM NaCl, 0.3% Tween-20; pH 7.5) at RT. Non-specific binding was blocked in freshly prepared blocking buffer (1% blocking reagent from Roche in wash buffer) for 30 minutes at 37°C. Sections were incubated for 1 hr at RT with anti-DIG-Fab-AP conjugate (diluted 1:750) in blocking buffer. Sections were washed three times for 5 min each in wash buffer. Sections were first equilibrated for 10 minutes in detection buffer (100 mM NaCl, 100 mM Tris-HCl pH 9.5, 50 mM MgCl2) and incubated in substrate solution (337 μg/ml NBT, 175 μg/ml BCIP, 5 mM tetramisole; in detection buffer) in a humid chamber at RT in the dark. The reaction was continued for up to 96 hr and stopped by rinsing slides in Tris-EDTA buffer (10 mM Tris and 1 mM EDTA) followed by water. Sections were mounted in Gelmount.

## Results

**Figure 1:**
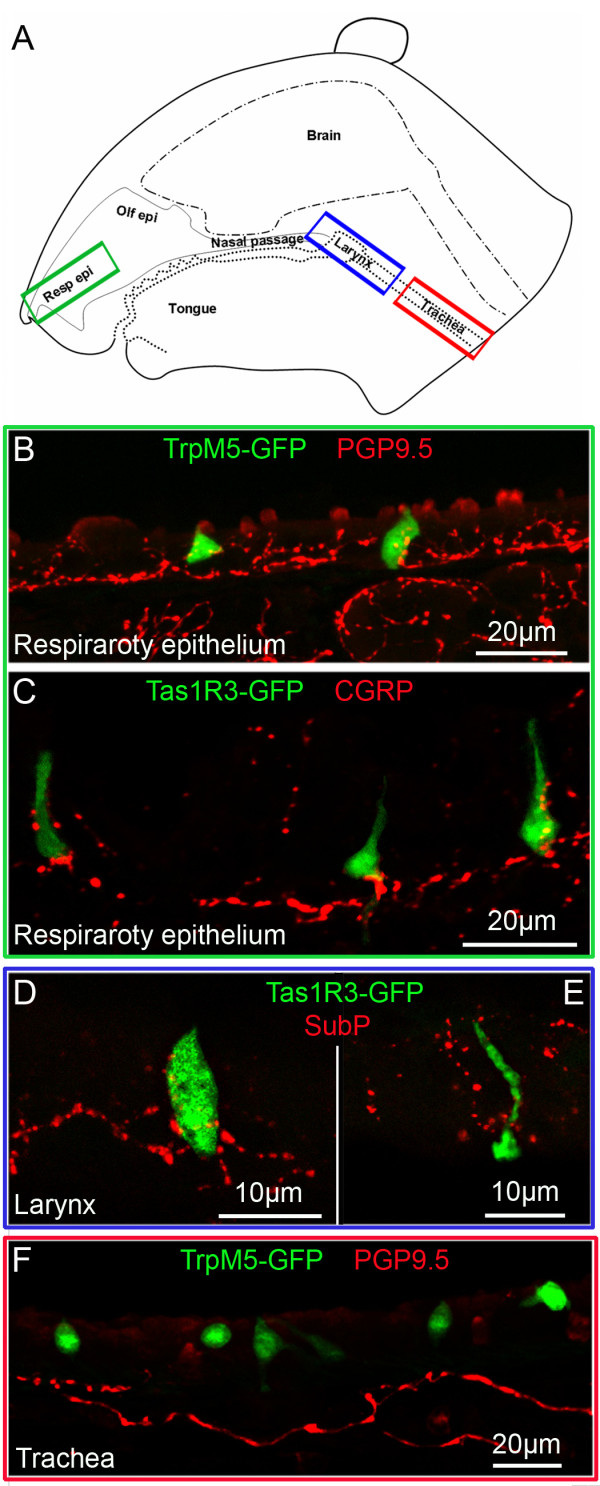
**Morphological relationships of SCCs in the airways**. **A**. Schematic representation of a sagittal section through the head of a mouse showing the airways and position of the different micrographs (green, blue and red rectangles). **B & C**. SCCs in the nasal respiratory epithelium. SCCs expressing TrpM5 or Tas1R3 (driven GFP) are intimately innervated by nerve fibers immunoreactive for PGP9.5 (B) or peptidergic nerve fibers of the trigeminal nerve immunoreactive for CGRP (C). **D & E**. In the larynx, Tas1R3-GFP+ SCCs are richly innervated by peptidergic (Substance P-immunoreactive) fibers probably from the superior laryngeal nerve (branch of the vagus) which shows responses to chemicals applied to the larynx [5,50]. **F**. SCCs of the trachea as shown by TRPM5-driven GFP are not closely embraced by nerve fibers although occasional en passant contacts can be observed.

**Figure 2:**
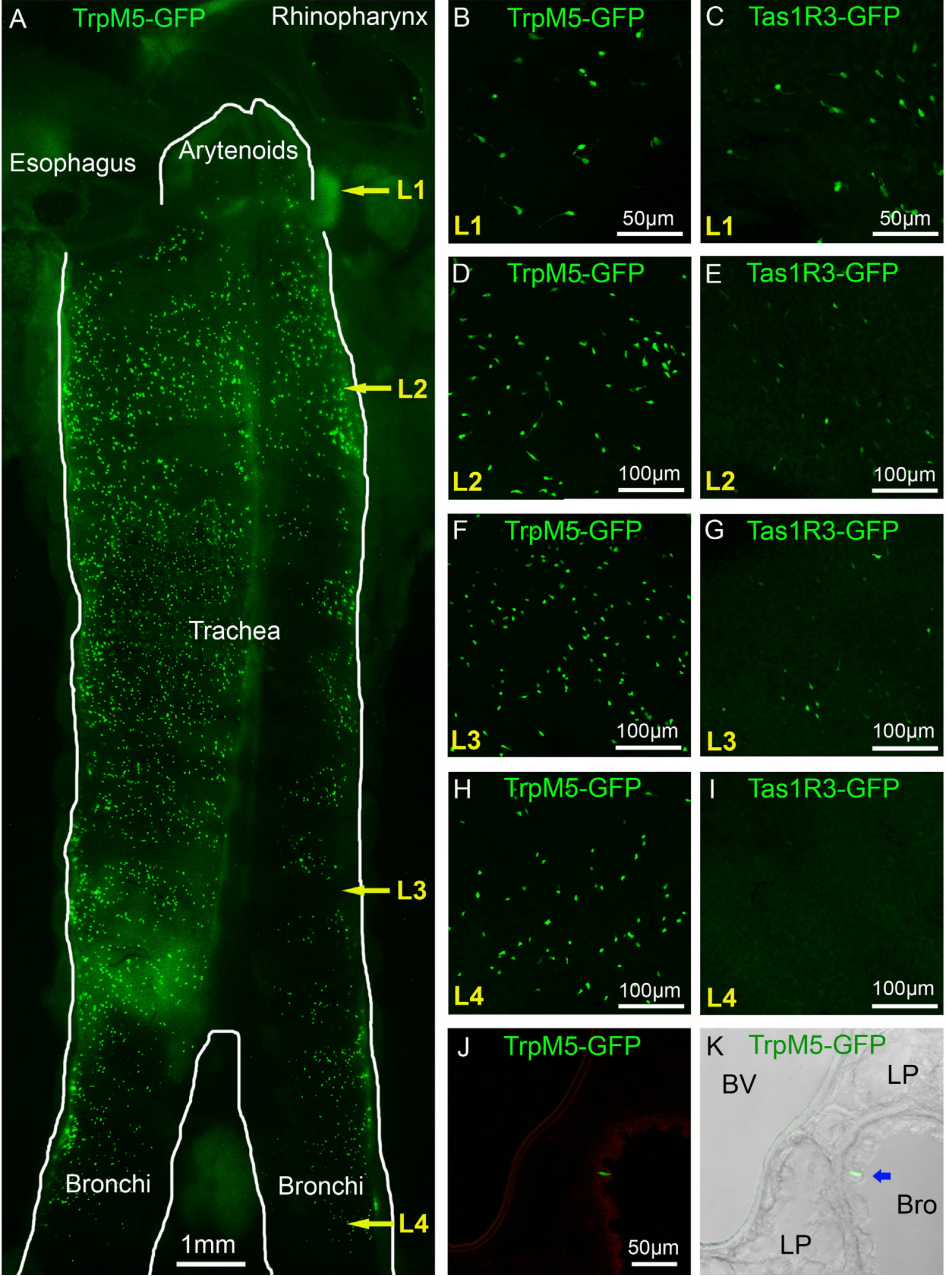
**SCCs are present to a different extent in the lower airways of TrpM5- and Tas1R3-GFP mice**. **A**. Micrograph of whole-mounted larynx and trachea opened laterally to show the distribution of the SCC cells (exhibiting green TrpM5-driven GFP fluorescence). Some SCCs are present in the hypoglossal portion of the larynx just below the arytenoids. Numerous TrpM5 GFP+ SCCs are present throughout the length of the trachea although at a lower density in the bronchi. L1, L2, L3, L4 refer to the levels of the micrographs of panels B-K of this figure. L1 = hypoglottis, L2 = proximal trachea, L3 = distal trachea, L4 = bronchi. B-I. Whole mount en face views of epithelium from different levels of the trachea in the two transgenic lines, TrpM5-GFP and Tas1R3-GFP **B. & C**. SCCs in the hypoglossal region. **D. - G**. Numerous SCCs are present in the proximal (L2) and distal (L3) portions of the trachea in the TrpM5-GFP line, while fewer are evident in the Tas1R3-GFP line. **H. & I**. SCCs are still evident in the bronchi of the TrpM5-GFP mouse while virtually none express Tas1R3-driven GFP. **J & K**. Tissue section through the lung showing SCCs in bronchioles of more than 400 ± 100 μm in diameter (I), but none in smaller bronchioles or alveoli**. J**. Green channel fluorescent image. **K**. Identical image field showing a Normarski image along with the fluorescence image. An SCC is indicated by a blue arrow in panel K. BV = blood vessel, LP = lung parenchyma, Bro = bronchioles.

In transgenic mice expressing GFP from either the Tas1R3 promoter or the TrpM5 promoter, we were able to identify SCCs in the airway mucosa (Figure 1B-F; Additional file 1). SCCs in the nasal respiratory epithelium are contacted repeatedly by peptidergic fibers of the trigeminal nerve (Figure 1B,C) and those in the larynx are closely associated with peptidergic fibers of that organ (Figure 1D,E). In contrast, the SCCs in the trachea of these mice are not densely innervated (Figure 1F), although in the hypoglossal portion of the larynx SCCs are innervated (Figure 1D,E). Numerous TrpM5 GFP+ SCCs are present throughout the length of the trachea approaching densities of 40-50 SCCs per 100 μm^2 ^(Figure 2A-B,D,F). SCCs are present in the bronchi, but at a much lower density (Figure 2H) than trachea. In the lungs, SCCs occur in bronchioles of more then 400 ± 100 μm in diameter (Figure 2J-K) but no SCCs are present in smaller bronchioles or in the alveoli. Although Tas1R3 GFP+ SCCs (including the laryngeal SCCs) are consistently present in the lower airways in mice (Figure 2 C,E,G), they are less numerous than TrpM5 GFP+ cells and are absent in the bronchi and more distally (Figure 2I).

**Figure 3:**
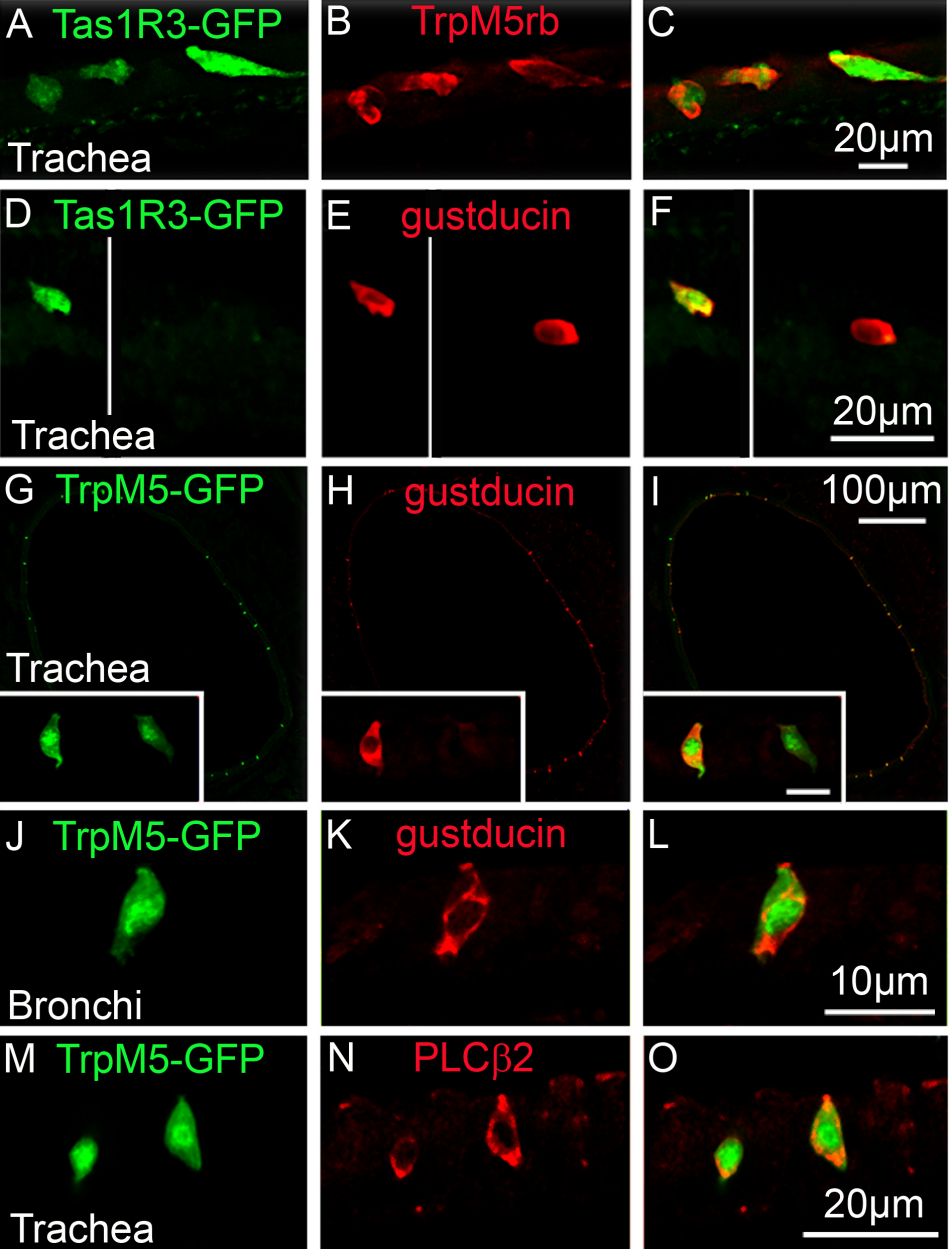
**SCCs in the lower airways express elements of the taste transduction cascade**. **A-F**. Single color channel (A & B; D & E) and merged (C & F) images of Tas1R3 GFP+ SCC cells in the trachea co-express TrpM5 and α-gustducin. **G-L**. Single color channel (G&H; J&K) and merged (I & L) images of TrpM5 GFP+ SCC cells in trachea (G-I) and bronchi (J-L) co-express α-gustducin. The insets of panels G-I show that α-gustducin is expressed only in a subset of the total TrpM5-GFP cell population (scale bar 10 μm for insets). **M-O**. Single color channel (M & N) and merged (O) images showing TrpM5 GFP+ SCC cells in the trachea co-express Plcβ2.

In all cases examined, Tas1R3 GFP+ SCCs co-express TrpM5 (Figure 3A-C,) and α-gustducin (Figure 3D-F). Conversely, not all the TrpM5 or α-gustducin immunoreactive SCCs exhibit Tas1R3-driven GFP expression, i.e. more SCCs express TrpM5 and gustducin than Tas1R3. In the trachea and bronchi, nearly all TrpM5-GFP expressing SCCs exhibit PLCβ2 immunoreactivity whereas fewer are immunoreactive for α-gustducin (Figure 3G-O).

**Figure 4:**
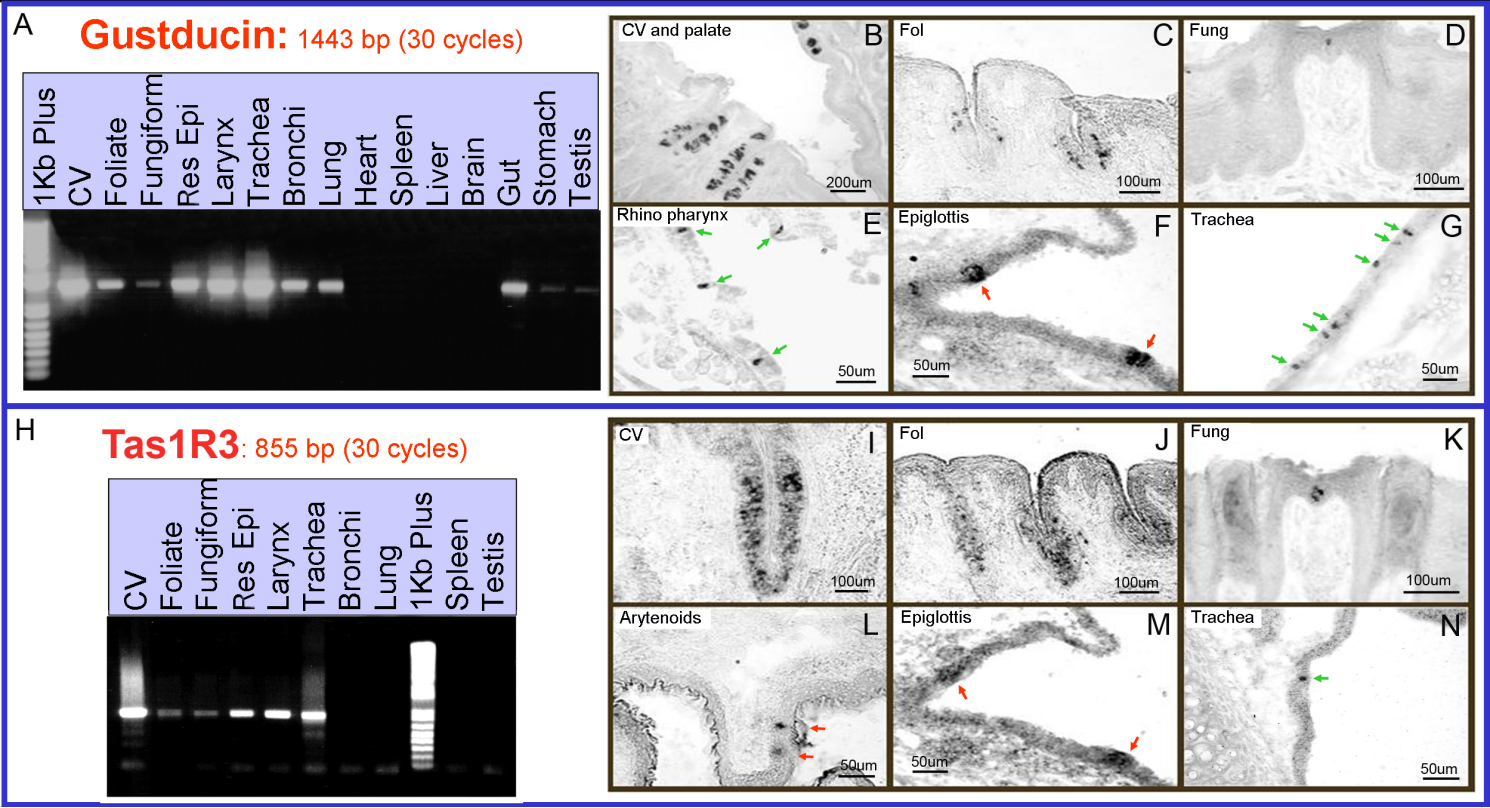
**Rat airways express the Tas1R3 receptor and the G-protein α-gustducin**. **A**. A PCR product for the α-gustducin gene is present in taste, respiratory and gastrointestinal tissue samples. **B-G**. Antisense probe (AS) for α-gustducin hybridizes with taste cells (B-D), as well as taste buds in the epiglottis (red arrows; F) and SCCs in the rhinopharynx and trachea (green arrows; E and G). **H**. The Tas1R3 gene PCR product is present in taste and airway tissue, but is not detectable in bronchi and lung. In contrast PCR for α-gustducin is positive in rat bronchi and lungs. Testis are positive for α-gustducin, but not for Tas1R3 **I-N**. The Tas1R3 AS probe hybridizes to taste tissue (I-K), laryngeal epithelium (red arrows indicate location of the taste buds; L-M) and a few SCCs in the trachea (green arrow; N). None of the negative control tissue (heart, spleen, liver and brain) showed expression of these two genes in PCR. Likewise, sense-control in situ probes showed no signal in any epithelium (Additional file 3). CV = circumvallate papillae; Fol = foliate papillae; Fung = fungiform papillae.

**Figure 5:**
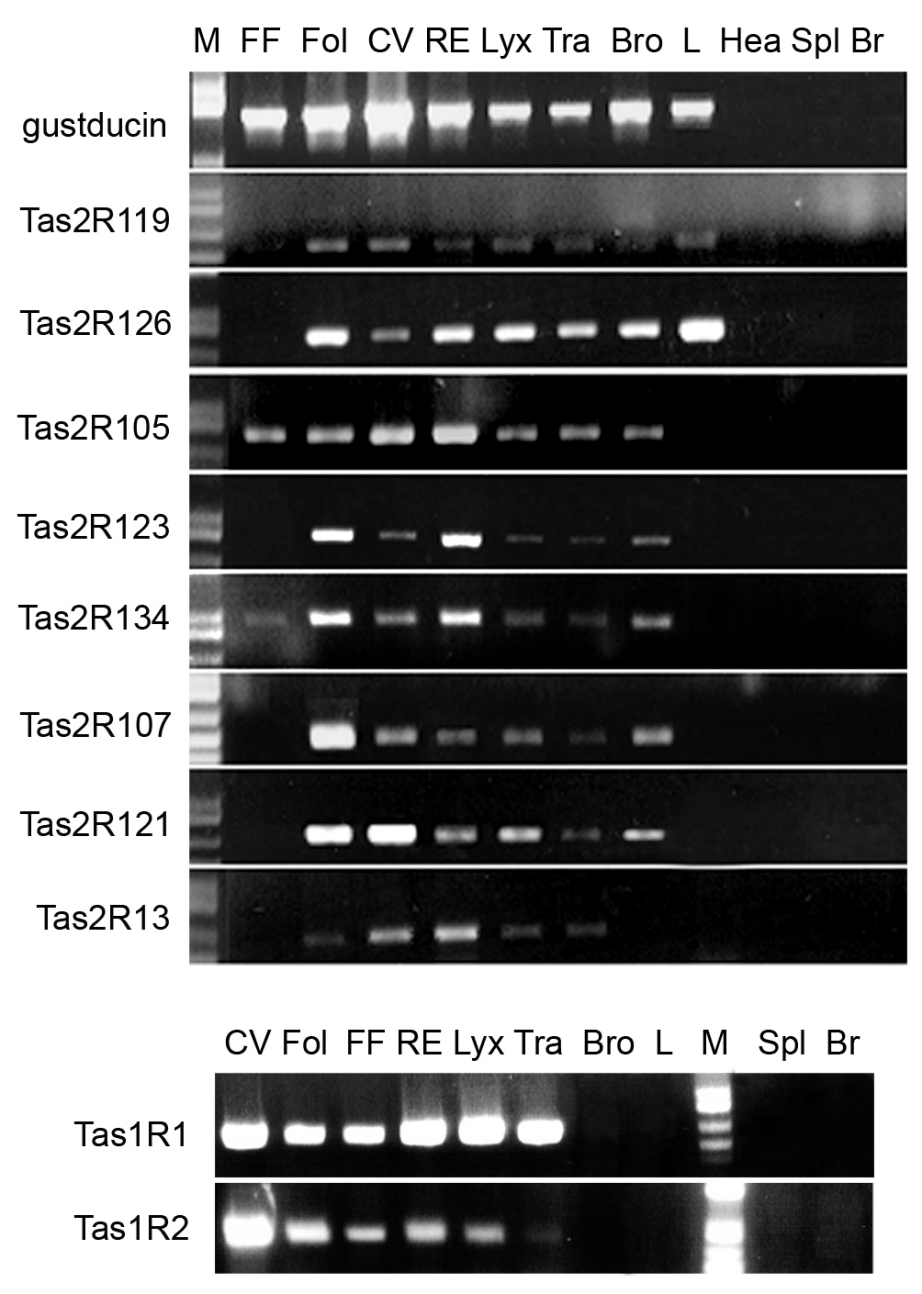
**Rat airways express members of the Tas1R receptor family and several Tas2R bitter receptors**. All of the Tas2R genes analyzed are expressed in the CV and Fol papillae, whereas only Tas2R105 and Tas2R134 are detected in the FF papillae. All tested Tas2Rs are detected in the nasal respiratory epithelium, larynx and trachea. With the exception of Tas2R13, all Tas2Rs are detected in the bronchi, but only Tas2R119 and Tas2R126 in the lung. None of the negative control tissue (heart, spleen and brain) showed any Tas2Rs or Tas1Rs expression. M = weight molecular marker; CV = circumvallate papillae; Fol = foliate papillae; FF = fungiform papillae; RE = respiratory epithelium; Lyx = larynx; Tra = trachea; Bro = bronchi; L = lung; Hea = heart; Spl = spleen; Br = brain.

To test whether other rodents show a similar distribution of SCCs and expression patterns of taste-related gene products, we examined the airways in rat. RT-PCR and ISH experiments confirmed the expression of Tas1Rs and several Tas2Rs in the rat airways (Figure 4-5). Antisense probe (AS) for α-gustducin hybridized with taste cells (Figure 4B-D,F) in tongue and epiglottis taste buds, as well as SCCs in rhino pharynx (Figure 4E) and trachea (Figure 4G). The Tas1R3 AS probe showed a consistent expression in the taste tissue (Figure 4I-K), in taste buds of the laryngeal epithelium (Figure 4L-M) and a few SCCs in the trachea (Figure 4N). Sense probes for these 2 genes yielded no specific reaction product (Additional file 3).

RT-PCR results for rat α-gustducin and Tas1R3 match the expression pattern observed in the ISH experiments (Figure 4A, H and Figure 5). Moreover all Tas1Rs were present in taste tissue and airway (Figure 4H and Figure 5). No Tas1Rs are detectable by PCR in bronchi and lung (Figure 4H and Figure 5) corresponding to the Tas1R3-driven GFP expression pattern described above for mouse (Figure 2I). In contrast, PCR for α-gustducin is positive in rat bronchi and lungs (Figure 4A and Figure 5), suggesting the presence of SCCs in the bronchioles in rats as we describe above for mice (Figure 2J-K).

The pattern of expression for several Tas2R bitter receptors is shown by the RT-PCR experiments in Figure 5. All of the Tas2Rs genes analyzed were expressed in the circumvallate and foliate papillae, whereas only Tas2R105 and Tas2R134 were detected in the fungiform papillae. In the nasal respiratory epithelium, larynx and trachea samples, all of the tested Tas2Rs were present. With the exception of Tas2R13, all tested Tas2Rs were present in the bronchial sample, although the Tas1R3 receptor was not expressed in the same sample. Of the tested Tas2Rs, only Tas2R119 and Tas2R126 were detectable in the lung sample. None of the negative control tissue (heart, spleen and brain) showed any Tas2Rs or Tas1Rs expression and control experiments were performed with primers for GAPDH using cDNA treated with and without the reverse transcriptase (RT) enzyme (Additional file 2).

In summary, SCCs are present throughout much of the airway, but with a higher density in upper regions. One or more Tas2R (bitter) taste receptors are expressed in all regions of the airway, but the Tas1R3 receptor tends to be expressed only in the upper airways.

## Discussion

Scattered chemoreceptive epithelial cells occur throughout much of the length of the airways, from the nasal respiratory epithelium to the bronchioles. Many SCCs, especially those in the upper airway, form contacts with the nervous system to transmit sensory information. SCCs were first described on the basis of morphology in the epidermis of aquatic vertebrates [19]. These cells were characterized as extending a tuft of microvilli to the surface of the epithelium and extending vertically through the height of the epithelium, to form distinctive contacts with nerve fibers. In fish, the SCCs appear to share some amino acid receptors with taste buds [29]. In the last decade, morphologically similar cells were described in the nasal epithelium of mammals [10,44] and were found to express many of the molecules of the taste transduction cascade including taste receptors, G-alpha-gustducin, PLCβ2 and TrpM5 [12]. The function of SCCs as chemosensory elements was suggested originally on the basis of morphology [19] and later confirmed by functional imaging studies [10,13,18,20,25]. Molecularly similar cells (sometimes called the diffuse chemosensory system; [45,46] are scattered throughout much of the respiratory and digestive epithelia. Yet not all cells that express taste-related transduction molecules should be considered to be SCCs in the original sense of the phrase.

In the nasal cavity, at least two types of SCCs exist, i.e. SCCs in the respiratory epithelium highly innervated by the trigeminal nerve and chemoreceptive microvillous SCC cells situated high in the main olfactory epithelium but not connecting to nerves [47-49]. Some TrpM5-GFP+ SCCs are observed in the nasopharyngeal epithelium (rhinopharynx in Figure 2A) and they are probably involved in chemoresponse to compounds refluxing into the nasal cavity from the mouth.

In the larynx, SCCs are present at epiglossal and hypoglossal levels with some taste buds in the epiglottis and arytenoids. The laryngeal SCCs lie in an epithelium innervated by the superior laryngeal nerve which responds to a variety of chemical stimuli [5,50]. Chemical activation of the superior laryngeal nerve evokes choking and other protective airway reflexes [1].

Within the trachea, many TrpM5+ epithelial cells are present. Some of the TrpM5+/gustducin+ cells in the trachea have stiff, parallel apical microvilli and constitute at least a subset of brush cells [14] which are defined by their tuft of stiff apical microvilli [28,51]. The tracheal TrpM5-positive cells of mice are sparsely innervated [20,52]. In contrast, similar TrpM5-positive SCCs of the upper airways (including nasal epithelium, vomeronasal duct, nasopharnx, hypoglossal portion of the larynx) are heavily invested with nerve fibers which can wrap around and repeatedly contact the SCC [10,13,18,20,25]; Figure 1). The differences in the pattern of innervation between SCCs of the upper airways and those in the trachea that we observe in mice may be species specific or may generalize to larger mammals including humans. Further study will be necessary in order to test this proposition.

In the bronchi and bronchioles, the SCCs are present but at a lower density than higher in the airway. No SCCs are found in smaller bronchioles or in the alveoli in mice. The higher number of SCCs in the upper airways implies higher sensitivity or a broader range of responsiveness in that region. Although few or no SCCs occur deep in the airways, chemical sensitivity may still be present due to direct chemoresponsiveness of free nerve endings distributed throughout the respiratory tree. The trachea and broncho-alveolar epithelium are innervated by the vagus nerve. Vagal Aδ and C fibres can directly respond to most irritants to elicit cough and bronchoconstriction responses to protect the airways [53-55]. What additional capabilities are added by the presence of SCCs higher in the respiratory tract is unclear.

Many nasal SCCs express members of the family of bitter taste receptors, the Tas2Rs [10]. Accordingly, many SCCs in the nasal respiratory epithelium and vomeronasal organ ducts respond to bitter-tasting ligands with an increase in intracellular Ca^2+ ^[13,20,25]. Intriguing is the presence of sweet/umami receptors (Tas1R family members) in the airways. The presence of this class of receptors was first indicated by Tas1R3-driven expression of a transgene in the nasal epithelium [40]. We confirm with both PCR and Tas1R3 transgenic animals that a subset of nasal SCCs expresses Tas1R3 as do some in the trachea. Whereas Tas2Rs in the nose detect bitter/toxic compounds as irritants [13,20,25], there are no reports of any chemosensitivity mediated by Tas1R receptors in the airways or any experiment that show the natural ligands for the Tas1R+ SCCs in the respiratory tract.

It is noteworthy that nasal SCCs co-express Tas1R3-WGA with Tas2R5 and Tas2R8 [40]. In contrast, receptor cells in taste buds express either members of the Tas1R or the Tas2R receptor families, but never both together [32]. Further, individual SCCs co-express Tas1R3 and α-gustducin. In the taste buds of the posterior part of the tongue, taste receptor cells that express Tas1R3 and Tas1R2 seldom express of α-gustducin [56]. The co-expression of Tas1R3 and gustducin does, however occur in taste buds of fungiform papillae and palate [57,58].

Chemosensory cells expressing elements of the taste transduction cascade are prevalent in both the gastrointestinal tract [12,59] as well as the respiratory tree [10,12,13,16,17,25,60,61]. Despite this similarity in molecular expression, these diverse chemoresponsive cells including SCCs and brush cells, are different in terms of function and therefore should not be considered to be a single cell type. Although the Tas2R/gustducin/TrpM5-expressing epithelial cells in the different tissues are similar in extending microvillous processes to the top of the epithelium and in utilizing similar receptor and transduction cascades, the downstream effects of cell activation are different. The SCCs in the nasal respiratory epithelium form synapses to evoke a neural response [20], whereas brush cells of the gut release peptides in a more paracrine fashion to modulate digestive activities [62].

The function of the SCCs in the lower airway still needs to be determined, but may involve local modulation of the airway epithelium (mucociliary clearance and secretory functions; [60]) or induction of a local response of the innate immune system. For example, tracheal SCCs are likely to respond to bacterial signalling molecules or other irritants as do nasal SCCs, but instead of communicating with nerve fibers, may release cytokines and other modulators locally to evoke responses in dendritic cells or macrophages. The SCCs of the nasal cavity, vomeronasal ducts and larynx hold crucial positions at the entrance to different respiratory organs. Thus, SCCs in these situations may be especially strongly connected to nerves that trigger protective reflexes of these respiratory organs.

Recently, three studies report Tas2R expression in the airways. The first study reports expression of Tas2R family members and related downstream signalling components by tracheal brush cells (solitary chemosensory cells in our terminology) in mice [52]. These investigators found that brush cells also express cholinergic traits and lie close to, or contact, subepithelial nerve fibers that express nicotinic acetylcholine receptors.

The second study examines human airway epithelium in vitro, reported that ciliated cells of respiratory epithelium express Tas2R bitter taste receptors and other downstream gustatory transduction components, e.g. gustducin. This study reported that the Tas2R molecules localized to the motile cilia and that those ciliated cells responded to bitter-tasting compounds with an increase in intracellular Ca^2+ ^and an increase in ciliary beat frequency [61]. Their results contrast with our observation in rodents that elements of the taste transduction cascade are present only in SCCs and not in other cell types of respiratory tract, including ciliated cells. This may reflect a species difference, or the use of an in vitro system in the Shah et al [61] study. The mere size difference in the airways between mice and humans is not the determining factor since the expression of gustducin in airway epithelium of another large mammal (i.e. cow), appears restricted to a distributed SCC population [11].

The third report shows Tas2R expression in airways describes the localization of taste transdcution components to cultured airway smooth muscle[63]. These investigators showed by PCR expression of Tas2Rs on human airway smooth muscle. We do not find evidence for expression of bitter taste transduction elements in the tracheal smooth muscle (as determined by immunocytochemistry or transgene expression in mice). Whether the presence of taste transduction components in smooth muscle of humans is again a function of the in vitro system or a species difference is unclear. The Deshpande et al [63] study also reports that stimulation of cultured smooth muscle cells with bitter compounds evoked increased intracellular calcium dependent on the bitter taste transduction cascade similar to the cellular activation we have reported in nasal SCCs [13,20,25]. Further, Deshpande et al report that, inhaled bitter tastants decreased airway obstruction in a mouse model of asthma. It is unclear from this report how the bitter ligands are envisioned to reach the airway smooth muscle which is covered by respiratory epithelum. We suggest that the in vivo responsiveness may have been attributable to activation of tracheal SCCs, which secondarily activate the airway smooth muscle. Members of the Tas2R family are expressed in the SCCs of the nasal and tracheal [52] respiratory epithelia and these SCCs respond to bitter compounds to evoke protective respiratory reflexes. Further study of both human tissue and animal models will be necessary to fully understand this system.

## Conclusions

In summary, we find that epithelial cells specialized for chemoreception are distributed throughout much of the respiratory tree of rodents. The nasal cavity houses cytologically distinct SCCs that are intimately connected to sensory nerve fibers. In the lower airways, SCCs expressing similar transduction cascades include some brush cells with a distinctive tuft of stiff apical microvilli [52]. Further, the molecular repertoire of chemoreceptor proteins differs somewhat between upper and lower airways with the sweet/umami receptor subunit Tas1R3 being expressed in many in nasal SCCs and being largely absent in the SCCs of the lower airway whereas the Tas2R receptors are expressed throughout. The presence of Tas1Rs in the airways is intriguing since hitherto these molecules were considered only to mediate positive features of ingested foods, i.e. the presence of carbohydrates (sweet) or amino acids (umami). In the airways, these same receptors may be involved in detection of chemicals in foodstuffs which would be appetitive in the mouth or gut but which trigger protective reflexes in the airways.

## List of abbreviations

AP: Alkaline phosphatase; BCIP: 5-bromo-4-chloro-3-indolyl-phosphate CGRP: Calcitonin gene related peptide; GFP: green fluorescent protein; GPCRs: G protein coupled receptors; ISH: in situ hybridization; LSCM: confocal laser scanning microscope; NBT: nitro blue tetrazolium; OCT: Tissue-Tek embedding medium; PB: phosphate buffer; PBS: phosphate-buffered saline; PFA: Paraformaldehyde; PGP9.5: Protein gene product 9.5 of the ubiquitin carboxy-terminal hydrolase; Plcྞ2: phospholipase C beta2; RT: room temperature; RT-PCR: reverse transcriptase-polymerase chain reaction; SCCs: solitary chemosensory cells; SSC: saline-sodium citrate; SubP: Substance P peptide; Tas1R: taste receptor family 1; Tas2R: taste receptor family 2; TrpM5: transient receptor potential channel M5

## Competing interests

The authors declare that they have no competing interests.

## Authors' contributions

MT, AS and TEF participated in design and coordination of the study. MT and MC carried out the RT-PCR assays. MT performed all immunofluorescence and in situ hybridization experiments. Confocal microscopy was performed by MT. MT wrote the manuscript to which AS, MC and TEF added their contributions. All authors read and approved the final manuscript.

## Pre-publication history

The pre-publication history for this paper can be accessed here:

http://www.biomedcentral.com/1471-2466/11/3/prepub

## Supplementary Material

Additional file 1 **In wild type mice no significant autofluorescence is apparent**. **A & C**. SCCs in the nasal respiratory epithelium (A) and trachea (B) immunoreactive for α-gustducin. **C & D**. The same epithelia shown in the green channel lack any autofluorescence, validating the GFP expression of the transgenic mice used to identify the SCCs. Click here for file

Additional file 2 **PCR experiments conducted with primers for GAPDH on cDNAs treated with and without the reverse transcriptase (RT) enzyme**. The constitutive gene GAPDH is present in all the templates obtained by adding the RT enzyme during the production of the cDNA (+RT, upper line), whereas it is absent in all templates not treated with RT enzyme (-RT, lower line). PCR product length is 273 bp. M = weight molecular marker; FF = fungiform papillae; Fol = foliate papillae; CV = circumvallate papillae; RE = respiratory epithelium; Lyx = larynx; Tra = trachea; Bro = bronchi; L = lung; Hea = heart; Spl = spleen; Liv = liver; Br = brain; Sto = stomach; Tes = testis. Click here for file

Additional file 3 **In situ hybridization using sense-control probes showed no signal in any epithelium**. Both sense probes for α-gustducin (A) and Tas1R3 (B) show no staining in the epiglottis as well as in other tissue (not shown). The blue arrows indicate the location of laryngeal taste buds circled by dotted lines. Click here for file
