# Supplementary figures and images for: Expression of taste receptors in Solitary Chemosensory Cells of rodent airways
Source: BMC Pulm Med. 2011 Jan 13;11:3. doi: 10.1186/1471-2466-11-3 (PMC3031280; doi:10.1186/1471-2466-11-3)

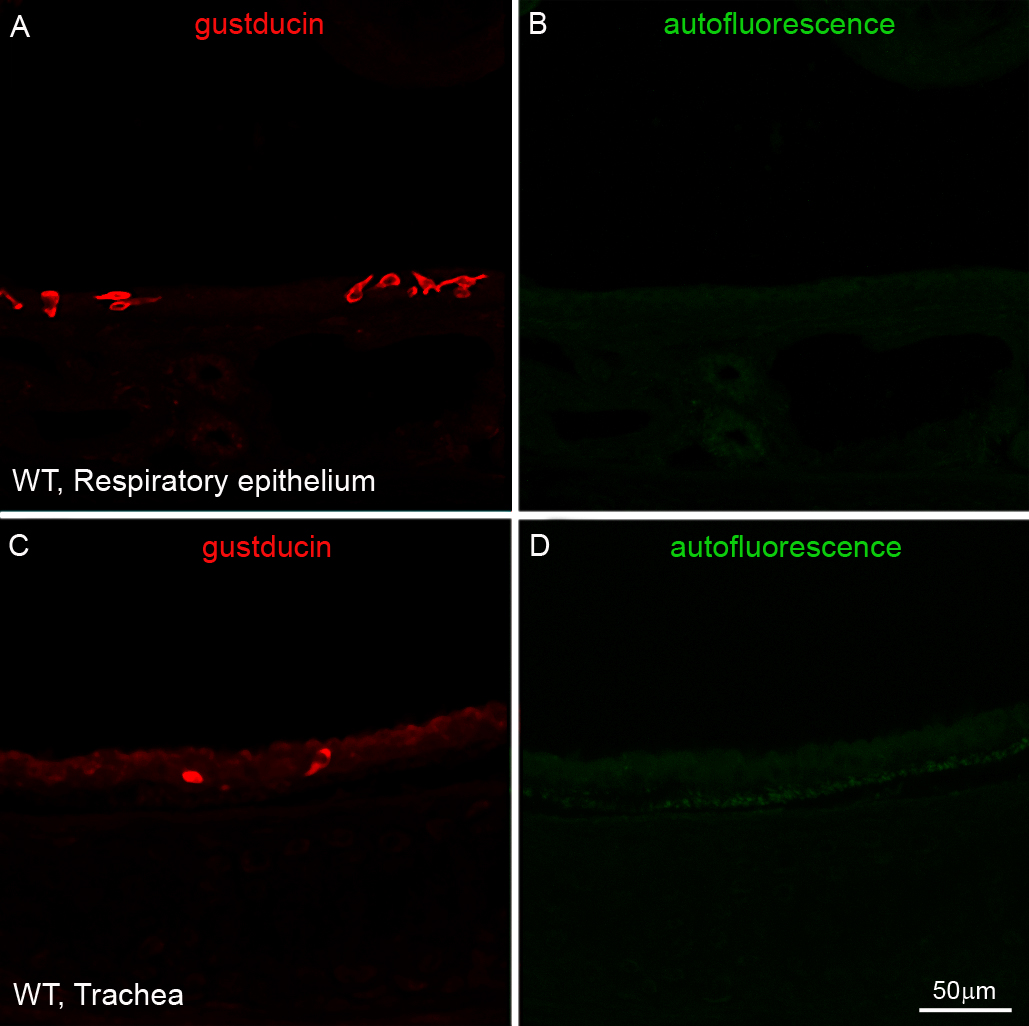

Supplement: Additional file 1 — In wild type mice no significant autofluorescence is apparent. A & C. SCCs in the nasal respiratory epithelium (A) and trachea (B) immunoreactive for α-gustducin. C & D. The same epithelia shown in the green channel lack any autofluorescence, validating the GFP expression of the transgenic mice used to identify the SCCs. [file 1471-2466-11-3-S1.JPEG]

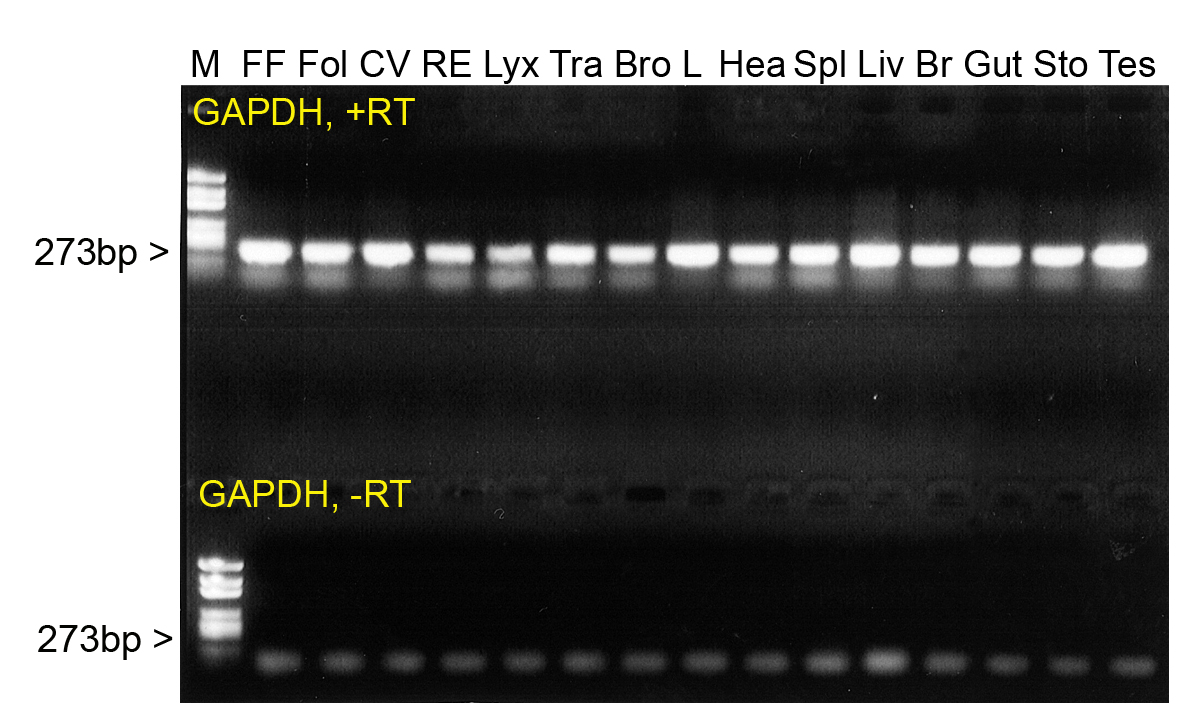

Supplement: Additional file 2 — PCR experiments conducted with primers for GAPDH on cDNAs treated with and without the reverse transcriptase (RT) enzyme. The constitutive gene GAPDH is present in all the templates obtained by adding the RT enzyme during the production of the cDNA (+RT, upper line), whereas it is absent in all templates not treated with RT enzyme (-RT, lower line). PCR product length is 273 bp. M = weight molecular marker; FF = fungiform papillae; Fol = foliate papillae; CV = circumvallate papillae; RE = respiratory epithelium; Lyx = larynx; Tra = trachea; Bro = bronchi; L = lung; Hea = heart; Spl = spleen; Liv = liver; Br = brain; Sto = stomach; Tes = testis. [file 1471-2466-11-3-S2.JPEG]

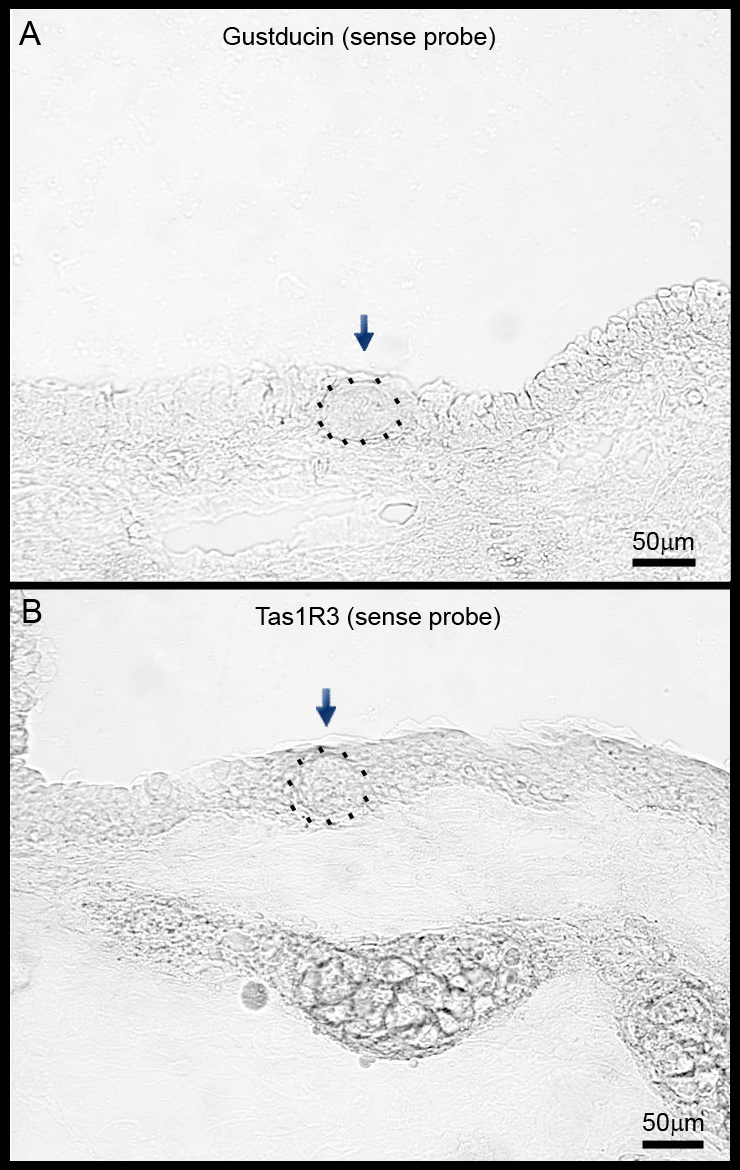

Supplement: Additional file 3 — In situ hybridization using sense-control probes showed no signal in any epithelium. Both sense probes for α-gustducin (A) and Tas1R3 (B) show no staining in the epiglottis as well as in other tissue (not shown). The blue arrows indicate the location of laryngeal taste buds circled by dotted lines. [file 1471-2466-11-3-S3.JPEG]
